# Batch and semi-continuous treatment of cassava wastewater using microbial fuel cells and metataxonomic analysis

**DOI:** 10.1007/s00449-024-03025-0

**Published:** 2024-06-06

**Authors:** Juan Carlos Quintero-Díaz, Jorge Omar Gil-Posada

**Affiliations:** Department of Chemical Engineering, Universidad de Antioquia, Calle 70 No. 52-21, Medellín, 050010 Antioquia Colombia

**Keywords:** Cassava wastewater, Bioelectricity, Anaerobic sludge, Bioelectrochemistry

## Abstract

The treatment of agroindustrial wastewater using microbial fuel cells (MFCs) is a technological strategy to harness its chemical energy while simultaneously purifying the water. This manuscript investigates the organic load effect as chemical oxygen demand (COD) on the production of electricity during the treatment of cassava wastewater by means of a dual-chamber microbial fuel cell in batch mode. Additionally, specific conditions were selected to evaluate the semi-continuous operational mode. The dynamics of microbial communities on the graphite anode were also investigated. The maximum power density delivered by the batch MFC (656.4 μW m^-2^) was achieved at the highest evaluated organic load (6.8 g COD L^-1^). Similarly, the largest COD removal efficiency (61.9%) was reached at the lowest organic load (1.17 g COD L^-1^). Cyanide degradation percentages (50–70%) were achieved across treatments. The semi-continuous operation of the MFC for 2 months revealed that the voltage across the cell is dependent on the supply or suspension of the organic load feed. The electrode polarization resistance was observed to decreases over time, possibly due to the enrichment of the anode with electrogenic microbial communities. A metataxonomic analysis revealed a significant increase in bacteria from the *phylum Firmicutes*, primarily of the genus *Enterococcus*.

## Introduction

Cassava (Manihot esculenta) is an important crop for the bioeconomy because of its rich starch content and usefulness as feedstocks for bioethanol, biochemicals, bioplastics, and other industries [1, 2]. The competitive advantage of cassava over similar crops hinges on its ability to grow well in acidic soils, thrive with limited rainfall or even survive during long periods of drought. With a net production of 2.5 million tons per year by 2020, Colombia, the third largest producer of cassava in Latin America, destined 10% of its national production to fulfill its demand for native starch (fresh flour) and fermented starch (sour starch). Cassava-based processing industries are typically characterized by their generation of large amounts of wastewater with high concentrations of organic matter, hydrogen cyanide and cyanohydrin. Cyanide compounds are toxic and a constant source of concern as they adversely affect soil chemistry, organisms, and plants when improperly disposed [3]. The cassava industry generates significant volumes of wastewater; for instance, 5 to 11 m^3^ of wastewater are produced during the processing of one ton of cassava roots into flour; Similarly, during the production of one tonne of cassava starch, 20 to 60 m^3^ of wastewater could be generated [4, 5]. The effluents that result during the production of both sour starch and fresh flour are discharged without any treatment. This situation is common place especially with small and medium-sized Colombian factories, known as “rallanderías”. At a large scale, cassava starch industries could produce up to 350 tons of starch per day and 5000 m^3^ d^-1^ of cassava wastewater which is well known for its high organic load (3.4–11.8 g COD L^-1^) and presence of cyanide ions (5.8–22 mg L^-1^) [6].

It has been shown that the reduction of cyanide and organic matter from cassava wastewater can be achieved by using either anaerobic or aerobic treatments [7–9]; however, its chemical energy, estimated as 12.24–42.48 kWh m^-3^, is hardly used [10]. Microbial fuel cells (MFC) are bioelectrochemical systems that harvest energy from organic waste sources [11, 12]. Due to its operating stability, eco-friendliness, and electricity production, MFC technology is gaining increasing attention as a cost-effective solution for water treatment. However, significant developments are still required, primarily in its maintenance during extended operational periods, scaling, and the design of efficient and economical materials [13, 14].

The performance of an MFC varies in response to changes in COD concentration and biomass density at the anode among other factors [15]. According to the literature, maximum power densities ranging from 13.8 and 1270 mW m^-2^ have been achieved, with total COD removal efficiencies ranging from between 22.8% and 92.1% [13, 14, 16]. The cassava wastewater treatment by using MFC technology has been poorly studied. However, recent findings report power density values ranging from 3.6 to 1800 mW m^-2^ with a COD removal of 53–90% have been reported [17–20]. It has also been reported that cyanide reduction of up to 70% has been achieved by using selected microorganisms that originate from the same cassava wastewater [17]. Finally, the use of anaerobic sludge has not shown any significant cyanide reduction [21].

Different types of microbial communities have been analyzed from natural sources such as aquatic sediments, soil samples, human gut microflora, as well as a wide range of urban and industrial wastewater sources, revealing diverse electroactive species primarily from the genera *Clostridium, Enterococcus, Geobacter, Shewanella*, among others [1]. The presence of microbial species such as *delta-Proteobacteria*, *Klebsiella oxytoca* and *Pseudomonas aeruginosa* have been confirmed from cassava wastewater samples and it is known that these organisms can generate electrical current, which make them suitable for MFC technology [17]. Significant changes have also been observed in microbial species communities when used with bioelectrochemical systems [2, 3]. However, no literature has been found regarding the variation of microbial species in sludge exposed to cassava wastewater, nor the long-term behavior of these microorganisms on MFC have been investigated.

This manuscript aims to evaluate the impact of organic loading on cassava wastewater treatment with MFC in batch operation, employing anaerobic sludge obtained from a local wastewater treatment plant. Additionally, semi-continuous MFC operation was utilized to assess electricity production performance and the dynamics of microbial community changes in the sludge during a 60-day period under fed-batch mode conditions.

## Materials and methods

### Anaerobic sludge

The anaerobic sludge used in this work comes from the San Fernando wastewater treatment plant located in Medellín, Colombia. The sludge had a biomass concentration of 14.6 g L^-1^ of volatile suspended solids (VSS) and a specific methanogenic activity of 49.8 mL CH_4_ g VSS^-1^  d^-1^. The sludge was stored at 4°C until its use. Sludge was centrifuged and washed two times with distilled water to remove residual soluble substrate before being used in the experiments [22].

### Cassava wastewater

In-house cassava wastewater was produced by adapting the protocol proposed by Alarcon and Dufour [23]. 1.5 kg of cassava roots were peeled, grinded and mixed with fresh water to obtain a total volume of 7.0 L. The mix was filtered and then allowed to be sedimented for 12 h. The sedimentation supernatant was taken as the cassava wastewater. Finally, a total volume of 6.0 L of cassava wastewater was obtained for every 1.5 kg of fresh cassava. The residual water thus produced was known to contain 6783 mg COD L^-1^, 3307 mg BOD L^-1^, 2177 mg TOC L^-1^, 6200 mg TS L^-1^ and 5.5 mg cyanide L^-1^. Chemical Oxygen Demand (COD), Biochemical Oxygen Demand (BOD), Total Organic Carbon (TOC) and Cyanide content, were determined using standard protocol of APHA [24].

### Microbial fuel cell

**Fig. 1:**
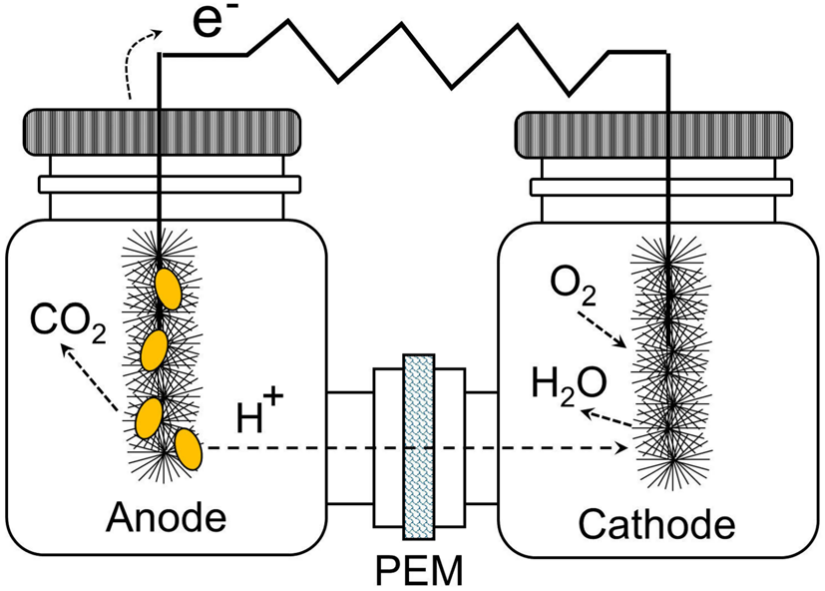
Dual chamber H-type microbial fuel cell basic construction (not to scale)

Experiments were conducted by duplicate on an standard dual chamber H-type microbial fuel cell, as the one sketched on Fig. 1. The experimental setup consisted of two glass chambers (anodic and cathodic compartments) of 250 mL each, separated by a proton exchange membrane (PEM) (Nafion 117TM, Sigma-Aldrich USA) with dimensions 1.5 cm $\times$ 1.5 cm. Graphite brush electrodes (2.5 cm of diameter, 2.5 cm of length) were used for both chambers. The MFC operating temperature was held constant at (30 ± 2)^∘^ C by using an in-house made temperature control chamber with capacity for hosting up to 4 MFC simultaneously.

Operating under anaerobic conditions, the anode chamber was loaded with a growth medium consisting of: 2.5 g L^-1^ NaHCO_3_, 0.1 g L^-1^ CaCl_2_ $\cdot$2 H_2_O, 0.1 g L^-1^ KCl, 1.5 g L^-1^ NH_4_Cl, 0.6 g L^-1^ NaH_2_PO_4_ $\cdot$H_2_O, 1.87 g L^-1^ Na_2_HPO_4_ $\cdot$12 H_2_O, 0.1 g L^-1^ NaCl, 0.1 g L^-1^ MgCl_2_ $\cdot$6 H_2_O, 0.1 g L^-1^ MgSO_4_ $\cdot$7 H_2_O, 0.005 g L^-1^ MnCl_2_ $\cdot$4 H_2_O, 0.001 g L^-1^ Na_2_MoO_4_ $\cdot$2 H_2_O, 0.05 g L^-1^ yeast extract and 33 mM of Methylene Blue. Cassava wastewater and the acclimatized mixed bacterial culture were added to obtain COD concentrations between 1.0 and 6.0 g L^-1^ of VSS. The anolyte was homogenised using a magnetic stirrer at 400 rpm for 72 h.

The cathode chamber was filled with 20 mM aqueous solution of Potassium ferricyanide (III). Oxygen was supplied through air bubbling using an aquarium pump at a constant flow. COD degradation, electricity production and electrochemical characterization of MFC was conducted by changing the initial charge of COD. Samples of anolyte were taken each 24 h, centrifuged to 5000 rpm for 20 min and used for COD and cyanide analysis.

In addition to the batch experiments, a semi-continuous operational mode was conducted in duplicate to evaluate the operational behavior of the cell over a 60-day period and to identify changes in the microbial community resulting from adaptation to electron transfer at the anode.

The cell was operated with cassava wastewater at a chemical oxygen demand (COD) of 6.8 g L^-1^ and a volatile suspended solids concentration of 6.0 g L^-1^. Every third day, 10% of the reactor volume was removed and replaced with fresh medium at the same initial COD concentration. On days 12, 30, and 48, the medium replacement was suspended for 6 days to evaluate the response of the device when the carbon source supply was limited. During the process, internal resistances of the cell (ohmic resistance and anode polarization resistance) were calculated by using the current interruption method to observe changes in electrochemical behavior throughout the operation. Biofilm analysis growing over anode and membrane was made through SEM. Metataxonomic analysis was conducted on biomass samples from the anaerobic sludge used as inoculum at the beginning (time zero) and on the biofilm taken from the filaments of the brush anode at the end of the treatment (60 days) to assess the changes in microbial diversity generated during MFC operation.

### Electrochemical methods

After inoculation, microbial fuel cells were operated under a external constant load of 1000 ohms. Data acquisition and electrochemical measurements were performed by using a multi-channel potentiostat with FRA capabilities (MultiPalmSense4, Palmsens).

With the aim of understanding the electrochemical behavior of the cell, MFC internal resistance measurements were conducted by using two methods: The steady discharging method and the current interrupt method [25]. This last method was used in the 60 days trial to avoid interrupting the ongoing process for an extended period. The MFC internal resistance can be understood as the opposition to the flow of current within the MFC. Basically, there are two main components that impact the MFC internal resistance ($R_{{\text{i}}}$). The first component, known as the ohmic resistance ($R_{\Omega }$), arises from the movement of ions across the electrolyte and through the proton exchange membrane. The second component, known as polarization resistance ($R_{{\text{P}}}$), arises from the energy barrier to the flow of current at the electrodes. Essentially, The MFC internal resistance can be expressed as follows:

1 $$R_{{\text{i}}} = R_{\Omega } + R_{{\text{P}}}$$

The steady discharging method was used to determine the total internal resistance ($R_{{\text{i}}}$). The polarisation curves were performed by using a series of resistors (5000, 2000, 1000, 500, 390, and 100 ohms), so current and potential difference values were measured across the MFC [26]. The relationship between current and voltage follows the relationship:

2 $$E_{{{\text{emf}}}} = {\text{OCV}} + IR_{{\text{i}}}$$

Where ($E_{{{\text{emf}}}}$) represents the electromotive force (the potential generated across the cell), also known as cell voltage ($V_{{\text{c}}}$), OCV denotes the open cell voltage, and the term $IR_{{\text{i}}}$ represents the overall voltage drop across the cell. The slope of the $V_{{\text{c}}}-I$ curve in the linear region represents the total internal resistance ($R_{{\text{i}}}$).

The power density (*P*) of the MFC was calculated by considering the electrode’s area (*A*), the external resistance ($R_{{\text{e}}}$) and the potential difference across the cell ($V_{{\text{c}}}$) as indicated by the following expression:

3 $$P = \frac{{V_{{{\text{cell}}}}^{2} }}{{AR_{e} }}$$

The maximum power density of the MFC was obtained from the power density vs. current plot.

The current interrupt method consists in monitoring the potential difference across the MFC at the steady state conditions; then turning the current off by opening the circuit, and simultaneously, monitoring the change in voltage as a function of time. To analyze the data related to current interruption and evaluate internal resistance, the Randles equivalent circuit was employed to describe the interface. This model comprising an $R_{\Omega }$ resistor in series with a parallel combination of a capacitor $C_{{{\text{dl}}}}$ and polarization resistance $R_{{\text{P}}}$. The polarization resistance relates to the charge transfer kinetic, $C_{dl}$ is the double layer capacitance of the electrode and $R_{\Omega }$ is the ohmic resistances of the cell electrolyte and membrane. The output voltage of the microbial fuel cells can be described as Eq. (4) [27, 28].

4 $$E_{{{\text{emf}}}} = {\text{OCV}} - I(R_{\Omega } + R_{{\text{P}}} ) - IR_{{\text{P}}} \exp ( - t/\tau )$$

Where $\tau$= $R_{{\text{P}}}$ $C_{{{\text{dl}}}}$, is the discharge time constant of the cell.

By fitting the experimental data to Eq. (4) by using the least squares method, it is possible to determine the parameters of the electric circuit model.

Coulombic efficiency (CE) was determined by calculating the total amount of charge that was generated by the cell (electrons transferred to the anode, $(Q_{e})$, divided by the total amount of charge that corresponds to the electrolyte’s COD load removal obtained during the wastewater treatment, according to the following expression:

5 $${\text{CE}} = \frac{{\int_{{t_{0} }}^{{t_{f} }} I dt}}{{\Delta {\text{COD}}\, V_{{\text{A}}} M^{{ - 1}} n F}} \times 100$$

Where *I* is the current, *F* is Faraday’s constant (96458 C mol^-1^), $\Delta {\text{COD}}$ is the change in chemical oxygen demand, $V_{\text{A}}$ is the volume of medium in the anodic chamber, *M* is the molecular weight of oxygen, and *n* is the number of electrons produced per mole of substrate.

The voltage efficiency ($V_{\text{ef}}$) can be found as the operating voltage ($V_{c}$) with respect to the maximum theoretical voltage at zero current (*OCV*) according to the following expression:

6 $$\begin{aligned} V_{\text{ef}}=\frac{V_{c}}{\text{COD}}\times 100 \end{aligned}$$

### Analytical methods

Colorimetric tests based on Standard Methods 5220-D [29] were employed in COD analysis to quantify organic matter oxidation during the Microbial fuel cell treatment and the concentration of total cyanide was determined using a titrimetric method based on Standard Methods 4500-CN-B,C,D [24].

### Scanning electron microscopy

During MFC operation, the microbial consortium produces a complex biofilm (made up of sugars, proteins, bacterial cells, etc.) not only on the anode surface but also on the proton exchange membrane. Therefore, the graphite electrodes and proton exchange membranes from the cells operated for 60 days were cut and subsequently subjected to SEM analysis. Biofilm samples coming from the anode and PEM were placed on a graphite tape surface and then coated with a thin gold layer. Surface morphology was achieved by using a Denton Vacuum Desk IV device. Morphological images were taken by using a JEOL JSM 6490 LV Microscopy high vacuum by means of an X-ray microprobe EDX, INCA PentaFETx3 Oxford Instruments.

### Metatoxonomy analysis

A metataxonomic analysis was conducted on the anaerobic sludge from the cell operated for 60 days to evaluate changes in bacterial population diversity between the beginning (16SBIOT0) and the end of the treatment (16SBIOT60) after 60 days. Initial and final samples (the latter taken from the electrode fibers), were preserved in refrigeration (4^∘^ C) and sent to the lab for DNA extraction. The DNA was extracted by using the PowerSoil^®^ DNA Isolation Kit (QIAGEN Laboratories) according to the manufacturer’s instructions. At the end of the extraction process, DNA quantification was performed using the light absorption method at 260nm using the NanoDrop^TM^ 2000-Thermo Scientific^TM^ equipment. The obtained DNA samples were frozen at − 20^∘^ C for the microbial diversity analysis for bacteria.

The V3–V4 hypervariable regions of bacterial and archaeal 16 S rDNA gene were amplified with the primers Bakt-341F (5′-CCTACGGGNGGCWGCAG-3′) and Bakt-805R (5′-GACTACHVGGGTATCTAATCC-3′).

The deep sequencing was performed on the Illumina MiSeq platform, generating paired-end reads of 300 bases each. The reads were trimmed using a quality threshold of Q30, and singletons as well as sequences shorter than 200 bases were removed. Sequence quality analysis and classification were conducted by using the MOTHUR platform (version 1.44). The coverage analysis for the processed microbial diversity samples showed a coverage value of > 98%.

## Results and discussion

### Organic load effect

**Fig. 2:**
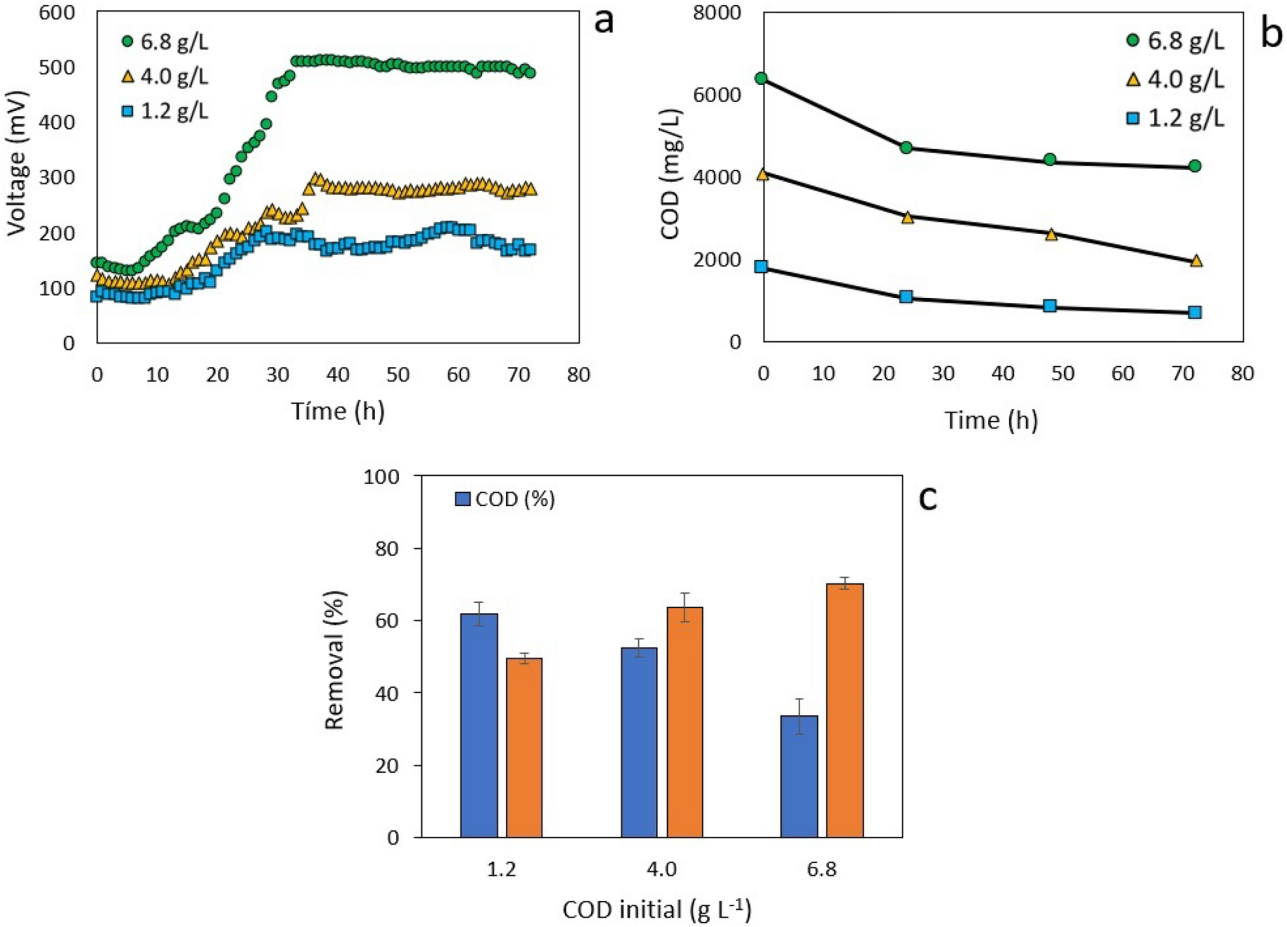
MFC voltage generation profile during the degradation of cassava wastewater (**a**), time domain COD profile (**b**) and COD and cyanide removal (**c**), as a function of the initial organic load

Aiming to evaluate bioelectricity production in a batch-mode operated MFC, samples of cassava wastewater were prepared at varying concentrations of organic substrate before undergoing a 72-hour treatment in the fuel cell. As can be seen from Fig. 2a, the voltage profile pattern obtained for each sample resembles microbial growth patterns with a latency phase lasting for 5 to 10 h, an exponential growth phase lasting for 10 to 35 h, and a stationary phase with 35 to 72 h duration. The first phase shows the sludge microbial consortium achieved rapid adaptation to the substrate; soon afterwards, the maximum voltage was not only rapidly established but also remained constant for the rest of the treatment.

As can be seen from Fig. 2b, the speed of consumption of organic matter reached its maximum value within first 24 h, which corresponds to the exponential voltage growth phase. In the stationary phase, however, a reduction in the speed of consumption of organic matter was found. The rapid initial COD removal can be explained by the possible presence of reducing sugars in the wastewater; these sugars are easily digested, broken down and absorbed by the microorganisms during the acidogenic metabolism. Once the concentration of reducing sugars is low enough so as the cassava starch becomes the main source of food for the microorganisms, then the rate of COD removal would slower down as the hydrolysis of starch is the rate limiting step for anaerobic digestion [30].

Acting as an electron donator during microbial metabolism, organic matter is crucial to MFC bioelectricity production. Under the experimental conditions considered here, it was found that voltage generation increases with increasing the initial organic load. Therefore, during the stationary phase, MFC voltage values of 190, 280 and 500 mV were reached for COD initial concentrations of 1.17, 4.0 and 6.8 g L^-1^ respectively. When the substrate concentration is increased, the conductivity of the medium is improved as a result of the enhanced ionic strength, attributed to the presence of organic acids produced during the acidogenic phase [31]. Therefore, the MFC internal resistance goes down while improving power generation [32]. In the present study, conductivity increased proportionally with the initial COD concentration from 3.1 mS at time zero to between 6.1 and 6.9 mS. This indicates the formation of ionic substances in the medium, which may correspond to organic acids.

Acetate is the most widely used substrate as a carbon source in electricity generation by Microbial Fuel Cells (MFC) [33, 34], and an increase in its concentration leads to an increment in power density [31]. However, it has been documented that elevated levels of acetate, serving as organic matter, can lead to a reduction in MFC voltage. This is attributed to the negative impact of excess substrate and accumulated loads on the efficiency of anaerobic microorganisms [32, 35]. This inhibition phenomenon has not been observed when using simple substrates such as glucose [36], but in line with our experimental results, MFC voltage generation increases with an increasing initial organic load without an observable inhibition phenomenon. As cassava wastewater is mainly composed of starch, then the generation of acetate molecules is restricted as their production is limited by the starch hydrolysis step. Similar findings have been reported by using other sources of starch such as potatoes and maize, where larger voltage generations have been achieved by increasing the organic loads; in the case of maize, however, when the organic load was too high, the current density went down, possibly due to substrate inhibition [37].

The organic content present in the cassava wastewater acts as an electron donor during the microbial metabolism, so COD reduction is expected from the production of current during the MFC operation. As shown in Fig. 2c, the removal of organic matter, expressed as COD, decreases with an increase in the initial organic content. In other words, the larger the initial COD, the lower the removal of organic content. After 72-h MFC treatment, the largest reduction of organic matter was 61.9% at an initial COD of 1.17 g L^-1^, compared to 33.5% reduction at 6.8 g L^-1^ of COD.

Due to its high cyanide content, cassava roots and products must be submitted to strict cyanide removal methods before commercialization and consumption. For the evaluated initial organic loads of COD in this set of batch experiments (1.2, 4.0, and 6.8 g L^-1^), the initial cyanide concentrations were (0.97, 3.2, and 5.5 mg L^-1^), respectively. As can be seen from Fig. 2c, the microbial sludge under consideration was capable to remove something like 50 to 70% of the initial cyanide. A control assay conducted at open circuit conditions (Initial cyanide concentration: 5.5 mg/L) exhibited a substantial level of degradation (53.3$\,\pm 3.5\%$). There are few studies on cyanide degradation in MFC systems [38]; however, it is known that cyanide can act as a nitrogen source for some microorganisms and be an electron donor [39]. Therefore, in the presence of electron acceptors with a higher redox potential, such as the anode, its degradation may be favored. This was evidenced by greater cyanide degradation observed in the cell assay compared to the open-circuit assay.

However, the degradation of cyanide is more dependent on microbial consortia than on the cell’s performance. For example up to 70% cyanide removal was achieved when an activated sludge coming from a wastewater plant was used to treat cassava effluents [17]. On the other hand, no significant cyanide degradation was registered when using an anaerobic sludge coming from a septic tank [21]. Certain microbial groups, specifically the bacterial phylum *Firmicutes* and the archaeal genus Methanosarcina, have been recognized as significant contributors to anaerobic cyanide degradation linked to methane production [40, 41]. These groups were the dominant species in the sludge samples used here, as showed in the subsequent meta-taxonomic analysis. The cyanide degradation pathway in anaerobic processes involves hydrolytic reactions that result in the production of HCOOH and NH_4_ as byproducts during cyanide hydrolysis [41, 42].

**Fig. 3:**
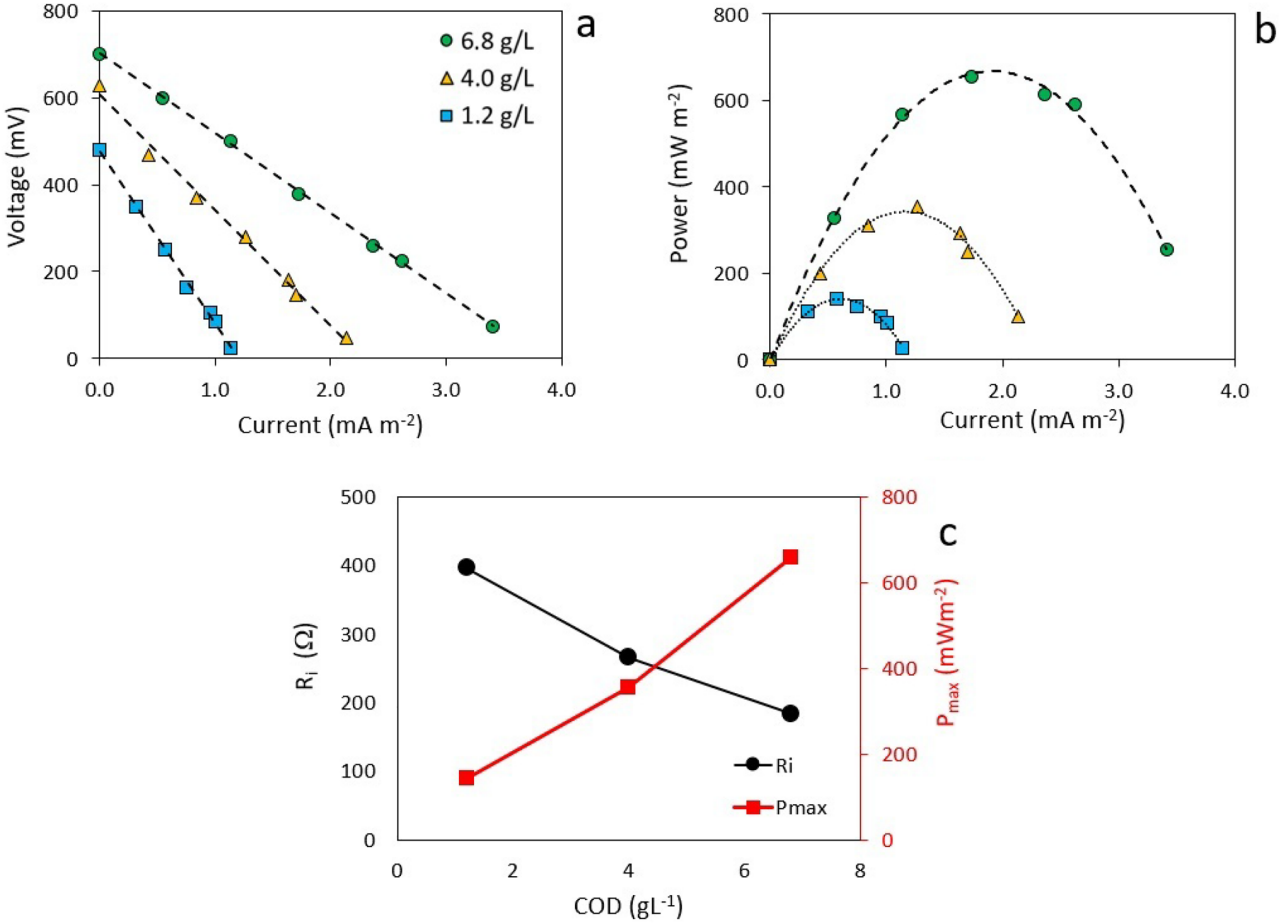
Electrochemical Characterization of the dual MFC as a function of the initial COD concentration. Polarization curves (**a**), power density curves (**b**), internal resistance, and power density (**c**), as a function of organic load

As depicted on Fig. 3a, the polarization curves reveal a strong linear association between the current density and voltage for all external resistances. Note the intercept at zero current density would correspond to the open circuit voltage; likewise, there is a negative correlation between the slopes of the COD vs. current with the initial organic load, this is, the greater the initial organic load, the less steep the slope, resulting in a smaller internal resistance. As can be seen from Fig. 3b, there is a positive correlation between power density and organic load. This is, as the initial organic load increases, the maximum power density generated by the MFC increases as well.

These behaviors are depicted more clearly in Fig. 3c. The higher the initial organic load, the smaller the internal resistance, and the larger the power generation. The presence of organic matter in the form of organic macromolecules allows the slow release of organic acids from microorganisms, thereby reducing the fermentative generation of acidic species that could decelerate cellular metabolism. On the other hand, higher concentrations of organic acids will increase the electrolyte’s conductivity, thus reducing both ohmic and internal resistances [32, 35].

As can be seen from Table 1, the largest the COD concentration, the largest the power density and the largest the electrical charge, so for a power density of 656.4 mW m^-2^ the corresponding charge was almost 101.0 A s. Meanwhile, the lowest internal resistance of the cell and the lowest organic matter degradation were determined at 183.8 $\Omega$ and 33.5%, respectively. Investigations in the treatment of cassava wastewater have revealed power density values comprised from between 3.6 and 1800 mW m^-2^ [17, 19]. The moderated power density values obtained in this work are possibly due to the use of carbon brush anodes with a reduced electro-active surface area, despite their high superficial area. Similar observations has also been made by other scientists when dealing with high-surface-area graphite brushes [19].

**Table 1:** Bioelectrochemistry characterization of MFC as a function of the initial organic load

COD (g L^-1^)	$Q_e$ (A s)	COD Removal (%)	EC (%)	$P_{\max }$ (m W m^-2^)	$R_{\text {int}}$ ($\Omega$)	V Efficiency (%)
6.8	101.0	33.5	1.6	656.4	183.8	66.7
4.0	51.0	52.5	1.5	353.8	265.1	44.4
1.17	40.6	61.9	0.6	142.0	395.9	37.5

Table 1 shows that the voltage efficiency decreases with decreasing the initial organic load. This reduction in efficiency is due to an increase in internal resistance, which generates potential drops that lower the thermodynamic potential during cell operation. The main reasons explaining this phenomenon are as follows: Firstly, the presence of electron acceptor species in the anolyte with a redox potential greater than that of glucose oxidation (− 428 mV), such as sulphates (− 220 mV), nitrates (421 mV), or even oxygen (820 mV), which comes from the cathode through the PEM, can compete for electrons at the anode [43]. Secondly, fermentative processes associated with anaerobic sludge can lead to the production of carbon dioxide and methane (− 240 mV) [44, 45]. Lastly, substrate consumption to produce biomass plays a significant role, where under aerobic conditions, the yield of biomass has been reported to range from 0.4 to 0.6 g of biomass per gram of organic substrate consumed. However, under anaerobic conditions, this value is much lower, reaching 0.05 to 0.15 g of biomass per gram of consumed organic substrate. For MFCs, this value is even lower, typically not exceeding 0.05 g of biomass per gram of consumed organic substrate [46].

The low production of biomass achieved with MFC’s is certainly a big competitive advantage when compared with both aerobic and anaerobic conventional methods. It is worth noting there is an inevitable conversion of some electron donors into biomass, and so there is a competence between the fermentation processes leading to the production of methane [45, 47].

It has been established that coulombic efficiency (CE) is a function of the microorganism type, substrate, mode of operation and type of MFC [48]. As indicated by Table 1, the higher the initial organic load, the larger the coulombic efficiency. This is, for initial organic loads of 1.17 and 6.8 g COD L^-1^, the corresponding coulombic efficiency values were 0.6 and 1.6%, respectively.

However, coulombic efficiency values ranging between 20% and 30% have been reported for similar initial concentrations of organic substrate. It is worth noting that to achieve such high values, researchers have utilized cassava wastewater with larger organic loads and subjected the anaerobic sludge to thermal treatment to reduce the population of methanogenic microorganisms [17, 18].

Importantly, when dealing with cassava wastewater, batch operation has been reported to render smaller CE values, in the order of 1.6%, when compared with continuous operation where CE values would reach 67% [16, 48, 49]. Moreover, as pointed out by recent publications, coulombic efficiency decreases with increasing external resistance. As was explained, the protocol implemented here uses an external resistance of 1000 $\Omega$, but maximal coulombic efficiencies have been reported with external resistances in the order of only 10 to 100 $\Omega$ [35, 50].

As was previously stated, there is a negative correlation between internal resistance and initial organic load. It has been reported, however, that a disparity between internal and external resistance renders a reduced power density generation [50]. From a practical point of view, parity between internal and external resistances must be achieved so the MFC would achieve its maximum power output. This condition can be fulfilled by using an adjustable resistor that allows to control external resistance as a function of the MFC parameters. Under the light of these findings, the initial organic load, in the form of COD, can be used as an indirect means to determine internal resistance and therefore, it can be used to adjust the MFC’s external resistance which will in turn render its maximum power output.

### Semi-continuous microbial fuel cell operation

**Fig. 4:**
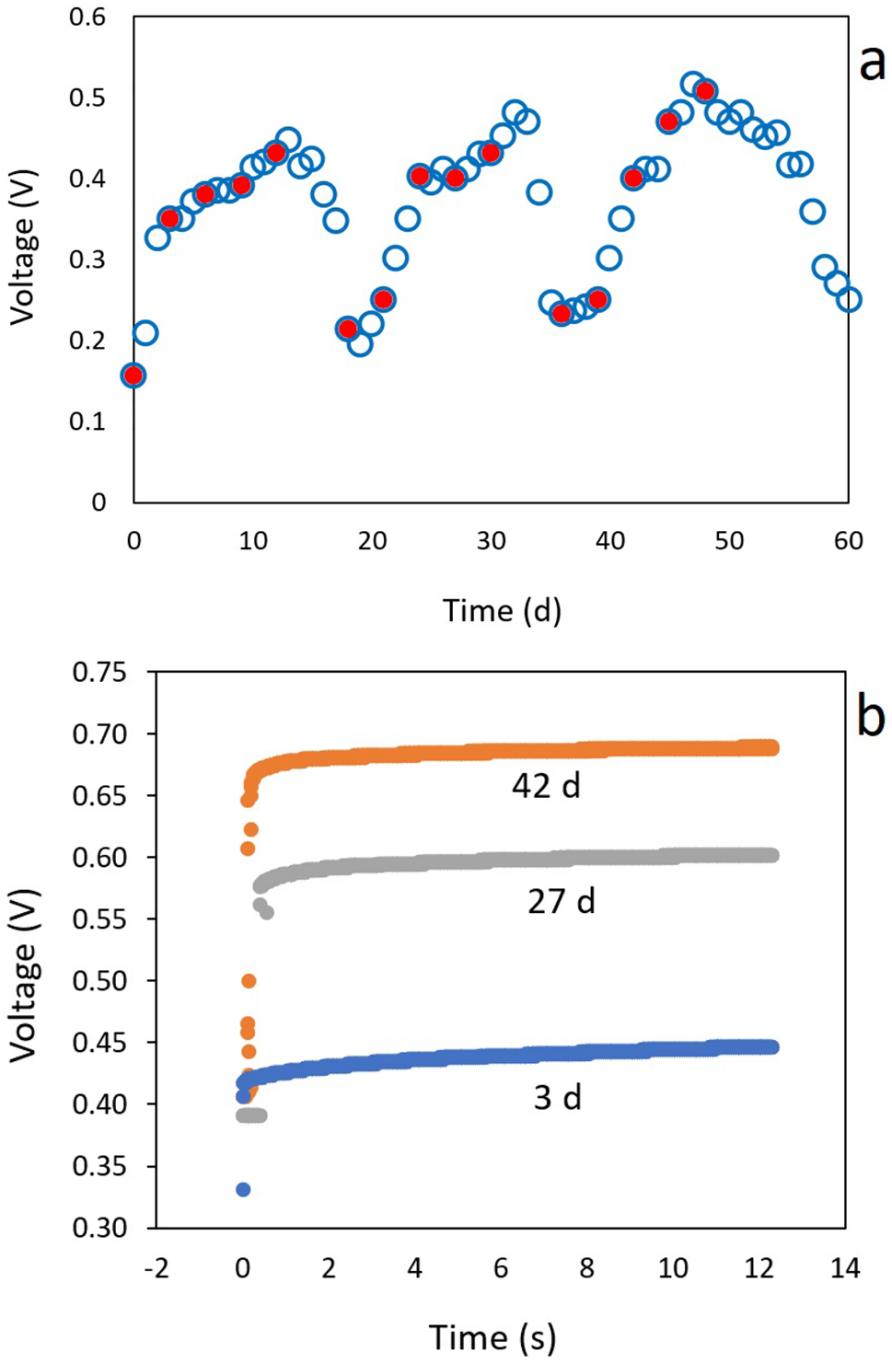
semi-continuous operation of dual chamber microbial fuel cell using cassava wastewater (**a**) and current interruption evaluations in three times during operation (**b**)

To evaluate the cell’s performance over an extended period, we conducted a 60-day operational trial with a semi-continuous MFC operation. Periodically, 10% of the depleted medium was replaced with fresh cassava wastewater, and both voltage and internal resistance were evaluated. As can be seen from Fig. 4a, the 60 day time domain voltage profile for the cell, shows a rapid increase in voltage during the initial days of operation, and thanks to the replacements of depleted medium (indicated with red markers), the voltage generation is maintained over time, reaching a peak of 447 mV after 13 days of operation. Between days 12 and 18, no replacements of depleted medium were performed, resulting in a rapid voltage drop to 192 mV. Once the replacement of depleted medium with fresh medium was resumed on day 18, a rapid voltage increment was observed, reaching a new maximum value of 470 mV on day 33. Between day 33 and 36, the replacements were suspended again, leading to a voltage decrease in the cell to 232 mV. The replacements and subsequent suspension of medium replacements were performed once more, showing a similar behavior, with a maximum voltage of 507 mV on day 48 and a minimum voltage of 250 mV on day 60.

From these results, it can be observed that the maximum and minimum voltage values obtained increase with the cell’s operation time, indicating a possible adaptation of the sludge to current production. Similar results have been reported for degradation of molasses with anaerobic river sediment sludge in dual-chamber microbial fuel cells (MFCs), where voltage increments and reductions were related to the organic matter accumulation or reduction over an 80 days period [51].

A recent study indicates the presence of organic matter and current production can be used for the development of biosensors. Anaerobic sludge was used to detect changes on biochemical oxygen Demand (BOD) with artificial wastewater. Basically, a gradual increase in peak voltage was evidenced over time during the accumulation and reduction of nutrients. The authors correlate this phenomenon with the enrichment of electrogenic communities at the anode [52].

During cell operation, measurements of internal resistance were performed using the current interruption method. The time domain voltage profiles, as can be seen from Fig. 4b, were fitted to Eq. (5) to obtain the values of ohmic resistance ($R_{\Omega }$) and electrode polarization resistance ($R_{p}$). As can be seen from Table 2, ohmic resistance increased over time from 160.1$\Omega$ to 191.0$\Omega$ between day 3 and day 42, while the electrode polarization resistance decreased from 256.4$\Omega$ to 203.0$\Omega$ over the same period. The rise in ohmic resistance can primarily be attributed to the expansion of the biofilm on the membrane surface, which limits mass transfer [53, 54]. This phenomenon results in a decrease in ion exchange capacity, ultimately leading to a reduction in power generation. [55, 56]. Based on the above, it is advisable to carry out periodic reactor stops to clean the membranes with a brush and distilled water, removing the biofilm [57].

**Fig. 5:**
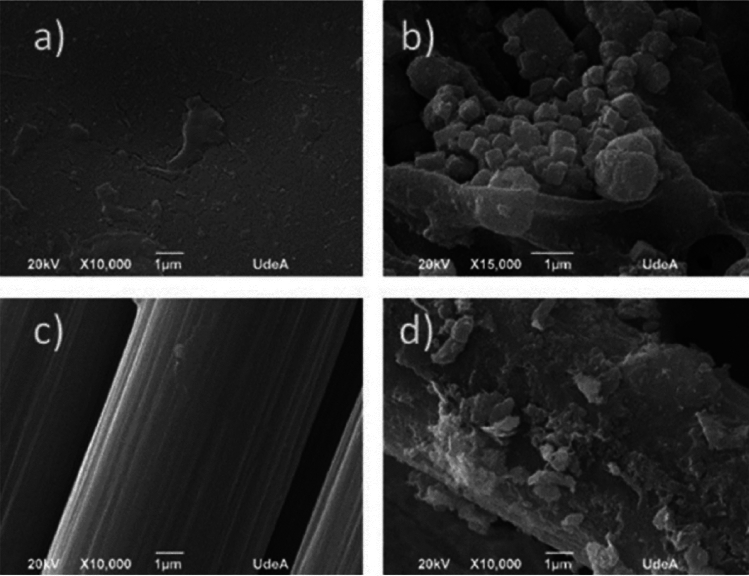
Biofilm formed at the Nafion 117 PEM membrane (top) and at the anode (bottom). **a** Clean membrane; **b** membrane after 60 days of treatment; **c** anode’s clean brush fibres; **d** anode after 60 days of treatment

Scanning electron microscopy inspection of both the PEM and the anode provided insights into their functioning. As shown in Fig. 5a, the membrane surface was clean and smooth before the treatment. However, after 60 days of treatment, a thick biofilm had developed on its surface, as demonstrated in Fig. 5b. A detailed observation reveals the presence of a biofilm composed of amorphous clumps that may contain bacteria and extracellular microbial by-products, such as biopolymers. Additionally, particles with defined cubic geometries were observed, possibly corresponding to the presence of inorganic salts [56, 58]. These elements induce deformations in the membrane on the surface of the anolyte.

Similarly, the initially clean surface of the anode, as seen in Fig. 5c, also developed a microbial biofilm after 60 days of operation, as revealed in Fig. 5d. The presence of individual bacteria and highly packed groups adhered to each other can be observed, along with the formation of clusters similar to those observed in the membrane, covering the entire surface of the electrode fibers. In these formations, the presence of cubic figures, as observed in the membrane, is not appreciated.

The reduction in polarization resistance can be explained by the potential increase in electrogenic communities at the anode, which was evident from the significant increase in the maximum voltage values obtained during cell operation. This aspect will be further analyzed with the results of the metataxonomic analysis shown next.

**Table 2:** Current interruption parameters by fitting data from Fig. 4b by using Eq. (5)

Operation time (d)	R_ohm_ ($\Omega$)	$R_{\text{p}}$ ($\Omega$)	$R_{\text{i}}$ ($\Omega$)	$D_{\text{dl}}$ (μF)	$r^2$
3	160.1	256.4	416.5	157.4	0.93
27	183.9	231.0	414.9	157.4	0.96
42	191.0	203.0	394.0	157.4	0.98

### Analysis of microbial species from the anode

The semi-continuous operation of the MFC allowed for the assessment of the sludge’s adaptability to electron transfer over the course of the 60-day operational period. During this period, the dynamics of voltage behavior over time were controlled by the addition or suspension of organic load and the values in the maximum voltage reached increased as time progressed. This might suggest that during this period, the sludge evolves in such manner that the anode becomes enriched with electrogenic species.

Samples of fresh anaerobic sludge and scrapings from the anode after 60 day operation were collected and used for microbial species analysis. An alpha diversity metataxonomic analysis was conducted by using the Illumina Miseq platform. A total of 262.998 raw-read pairs were generated out of 300 bases each. The readings were filtered out with a Q30 quality threshold and singletons. Sequences shorter than 200 bases were removed. Sequence quality and classification analyses were performed by using the MOTHUR platform.

The experiment generated a total of 132,702 raw-read pairs for the initial sample at time zero, named 16SBIOT0, and 130,296 raw-read pairs for the final sample named 16SBIOT60 (refer to Table 3). The coverage analysis for the processed samples of microbial diversity showed a coverage value $>98\%$, for all the samples indicating an appropriate sampling effort for capturing most of the bacterial diversity. The Shannon and Simpson diversity indicates that the collected samples exhibited high microbial diversity.

Based on the processed sequences, bacteria were dominant for both samples, accounting for 96.2% and 99.7% of the total count for the time zero and final sludge, respectively, while Archaea represented 3.70% and 0.27% for the time zero and final sludge respectively. The complete dataset revealed 38 bacterial phyla with 400 genera and three archaeal phyla with eight genera. The most abundant Archaeal phylum was *Halobacterota*, comprising 3.6% of the fresh sludge at time zero, and its abundance reduced to 0.19% in the treated sludge within the microbial fuel cell. Something like 10.3% and 1.7% of the processed sequences were classified as unknown phyla for the fresh and treated sludge samples respectively, while 49.7% and 22.3% of the processed sequences could not be assigned any taxonomic genera by the classifier.

**Fig. 6:**
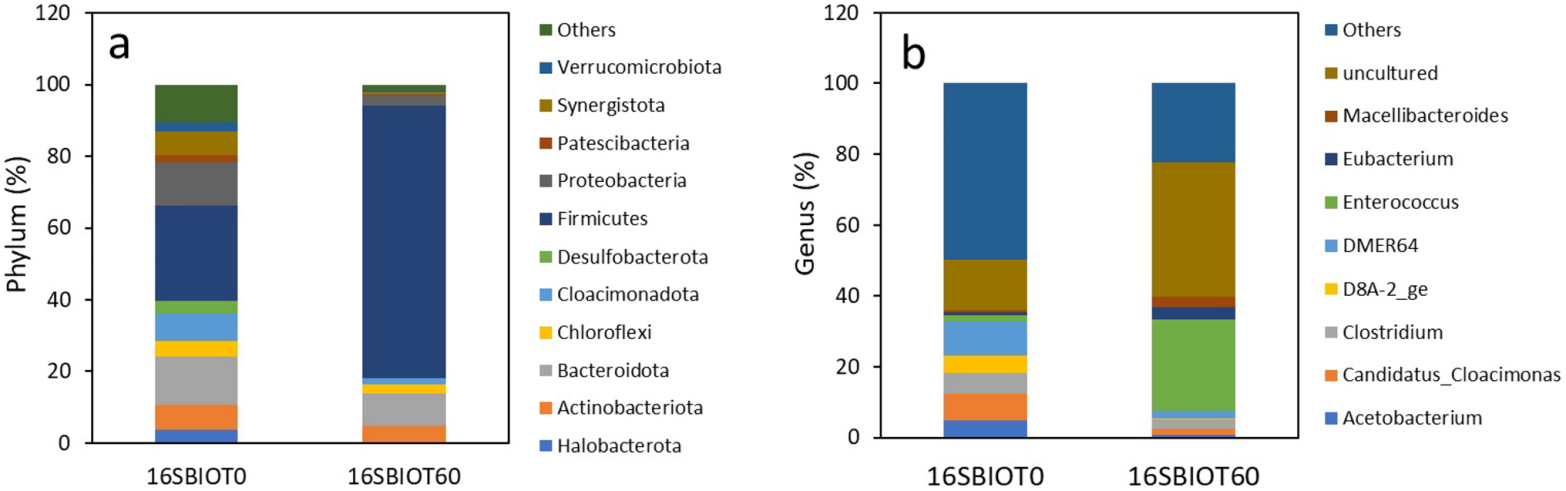
Relative abundance of prokaryotic on phylum level (**a**) and relative abundance on genus level (**b**), from the anaerobic sludge used during the operation of the microbial fuel cell, at the beginning of the operation (16SBIOT0) and at the end of the operation (16SBIOT60)

Ten types of bacterial phyla were observed, each at significant proportions across the samples. The population abundance of the different samples at the phylum level is shown in Fig. 6a. The three dominant phyla in the fresh sludge were *Firmicutes* (26.4%), *Bacteroidota* (13.4%), and *Proteobacteria* (12.3%), followed by *Cloacimonadota* (7.7%), *Actinobacteriota* (7.2%), *Synergistota* (6.7%), *Chloroflexi* (4.3%), *Halobacterota* (3.6%), *Desulfobacterota* (3.6%), *Verrucomicrobiota* (2.7%), *Patescibacteria* (1.9%), and *Others* (10.3%). On the other hand, in the final sludge, the dominant phyla were *Firmicutes* (75.9%), *Bacteroidota* (8.8%), and *Actinobacteriota* (4.7%), followed by *Proteobacteria* (2.8%), *Chloroflexi* (2.7%), *Cloacimonadota* (1.8%), *Synergistota* (0.5%), *Patescibacteria* (0.5%), *Halobacterota* (0.2%), *Desulfobacterota* (0.15%), *Verrucomicrobiota* (0.3%), and others (1.7%).

The only phylum that increased its proportion during the 60-day MFC process was the *Firmicutes*, rising from 26.4% to 75.9%. Meanwhile, the other phyla decreased in proportion, such as *Bacteroidota* and *Proteobacteria*. These results correlate with the richness and alpha diversity indices ACE, Shannon, and Simpson, which were higher in the sludge at zero time than in the 60-h sludge (refer to Table 3).

The phylogenetic composition revealed a distinct bacterial genus-level structure across the samples at time zero and at the end of the treatment, refer to Fig. 6b. Among the identified genera, the dominant ones from the fresh sludge were *DMER64* (26.9%), *Candidatus_Cloacimonas* (21.1%), *Clostridium* (15.7.3%), and *D8A2ge* (13.8%), followed by *Acetobacterium* (13.5%), *Enterococcus* (4.2%), *Eubacterium* (2.7%), and *Macellibacteroides* (2.1%). On the other hand, in the final sludge, the dominant genera were *Enterococcus* (64.9%), *Eubacterium* (8.6%), *Macellibacteroides* (7.6%), and *Clostridium* (6.4%), followed by *DMER64* (5.0%), *Candidatus_Cloacimonas* (4.4%), *Acetobacterium* (2.0%), and *D8A2ge* (1.2%). The genera *Enterococcus* and *Eubacterium* exhibited an important increase in their proportion during the operation of the cell.

**Table 3:** Alpha diversity analysis of anaerobic sludge samples at time zero and after 60 days of operation

Sample	Read count	Q30 (%)	nseqs	Coverage (%)	Observed (Sobs)	ACE	Shannon	Simpson
16SBIOT0	132702	87.0	54577	0.98	1819	4900.0	4.6	0.97
16SBIOT60	130296	87.7	53937	0.99	1162	3524.5	2.8	0.80

The most representative phyla found with anaerobic digestion plants correspond to *Proteobacteria*, *Bacteroidetes*, *Firmicutes*, and *Actinobacteria* [59], although their proportions vary depending on the combination of co-substrates present and the operational conditions of the reactors. These phyla correspond to the dominant groups in the sludges used in this study. Considering that *Firmicutes* and *Bacteroidota* form the most abundant group of hydrolytic bacteria [60], their prolonged cultivation in waters containing polysaccharides, such as cassava wastewater, explains the significant increase of these two groups in the sludges exposed to the MFC treatment for sixty days. On the other hand, the *Chloroflexi* phylum is composed of hydrolytic bacteria capable of degrading carbohydrates and acidogenic bacteria that produce acetate, while most bacteria in the *Proteobacteria* and *Actinobacteria* phyla act in the acidogenesis processes, converting organic matter into propionate and acetate [61–63].

The common bacterial genera in anaerobic sludges correspond to those observed in the fresh sludge used for the operation of the MFC. The genus *DMER64*, belonging to the *Bacteroidota* phylum, consists of hydrolytic bacteria that can also metabolize carbohydrates to produce acids and participate in electron transfer during methanogenesis [64]. On the other hand, bacteria of the genus *Candidatus_Cloacimonas* have the ability to ferment carbohydrates and to produce not only hydrogen but $CO_{2}$. The bacteria of the genera *Clostridium*, *D8A2ge*, *Acetobacterium*, *Enterococcus* and *Eubacterium* exhibit a wide metabolic diversity, ranging from hydrolytic capacity in *Clostridium*, carbohydrate fermentation in *Acetobacterium* and *Enterococcus*, to methane production in *Eubacterium*. This metabolic diversity suggests that the fresh sludge used in this study was adapted to treat complex wastewater, up to methane production.

The anaerobic sludge, after being developed for 60 days in a microbial fuel cell (MFC), underwent significant changes on its microbial diversity, shifting from 26% of bacteria belonging to the *Firmicutes* phylum reaching 75.9%, without any significant reduction on the number of observed individuals (Table 3). As mentioned earlier, the bacteria of the *Enterococcus* genus are mainly hydrolytic and fermentative, suggesting that the sludge increased its capacity to degrade polysaccharides and ferment sugars without losing the ability to produce methane. The *Proteobacteria* phylum is the bacterial group most frequently reported with electrogenic activity [65]. However, this phylum experienced a reduction from 12.3 to 2.8%. The relatively low abundance of *Proteobacteria* detected might suggest a limited contribution to the levels of metabolic activity in the electricity generation of the examined fuel cell. Other studies have also reported a significant reduction on the Proteobacteria phylum during acetate treatment in microbial fuel cells [66].

Other investigations exploring microbial community dynamics with microbial fuel cells over similar time periods, such as this manuscript, have yielded consistent findings. Notably, the prevailing detected phyla were *Firmicutes* and *Bacteroidota* [67–69].

The dominant genus of the phylum *Firmicutes* was *Enterococcus*, accounting for an abundance of 64.9% compared to other identified genera. Early studies, such as the one presented by Wrighton et al. [66], and more recent ones, have demonstrated the ability of *Firmicutes* isolates, such as the genus *Enterococcus* [70, 71], and the genus *Clostridium* [72, 73], to produce electricity. New studies will be required, involving isolations and electrochemical evaluations that confirm the presence and increased abundance of electrogenic microorganisms in the 60-day sludge, similar to what has been done in other microbial sources [72]. On the other hand, the relative abundance of *Eubacterium* indicates the sludge’s capacity to produce methane, which, as known, reduces the Coulombic efficiency in microbial fuel cells [74].

The production of electricity in a microbial fuel cell using wastewater from cassava processing is an alternative for its treatment and energy valorization. Additionally, it is effective in removing cyanide compounds. Regulating the organic matter content of cassava wastewater allows for efficient control of microbial fuel cell operation, enabling the development of strategies to maintain cell functionality over extended periods. This prolonged operation not only increases productivity but also enhances electrical power through the spontaneous selection of microbial species with enhanced electrogenic capabilities. Subsequent development of this technology includes the evaluation and optimization of cells in continuous operation and scaling up to achieve larger treatment volumes over time.

## Conclusions

During the treatment of cassava wastewater using anaerobic sludge with batch microbial fuel cells, an increase in energy production was observed as the organic load in the wastewater increased, while the rate of organic matter degradation was reduced. The highest electrical charge and power generated were achieved at an initial concentration of 6.8 g COD L^-1^, with values of 101.0 A$\cdot$s and 656.4 mW m^-2^, respectively. This behavior is attributed to the reduction in the internal resistance of the cell that took place when the initial organic load was increased. The anaerobic sludges used demonstrated the ability to remove cyanide from cassava wastewater, with removal percentages ranging from 50% to 70%. The cyanide treatment in an MFC generates a significant increase in degradation. The presence of bacterial phylum *Firmicutes* and the archaeal genus *Methanosarcina*, known for their cyanide degradation capabilities, were likely responsible for this degradation.

The semi-continuous MFC behavior significantly depends on the organic load, functioning similar to a biosensor where current is generated as response to the presence of organic matter. Over the course of operation, ohmic resistance increases, possibly due to membrane fouling, while polarization resistance decreases, probably due to electrode enrichment with electrogenic bacteria. Metataxonomic analysis of the sludge and the biofilm formed on the electrode after 60 days revealed a significant increase in *Firmicutes* bacteria, increasing from 26.4 to 75.9%. From this phylum, the dominant bacteria was the *Enterococcus*, which, along with bacteria from the *Clostridium* genus, also present, have been reported as electrogenic.

Microbial fuel cells can convert complex wastewater, such as cassava processing-derived effluent, into electricity production. It’s possible to control the performance by adjusting the organic matter concentration to develop prolonged operation times. Additionally, biomass enrichment is achievable. However, the obstruction of the PEM can reduce its performance, necessitating periodic maintenance for stable operation.

## Data Availability

Not applicable
